# Supplementary material for: Effective screening of SARS-CoV-2 neutralizing antibodies in patient serum using lentivirus particles pseudotyped with SARS-CoV-2 spike glycoprotein
Source: Sci Rep. 2020 Nov 5;10:19076. doi: 10.1038/s41598-020-76135-w (PMC7645753; doi:10.1038/s41598-020-76135-w)
Supplement: Supplementary file 1 — Supplementary Information. [file 41598_2020_76135_MOESM1_ESM.docx]

**Effective Screening of SARS-CoV-2 Neutralizing Antibodies in Patient Serum using Lentivirus Particles Pseudotyped with SARS-CoV-2 Spike Glycoprotein**

**Supplemental Material**

^1*^Ritesh Tandon, ^1^Dipanwita Mitra, ^1^Poonam Sharma, ^1^Martin G. McCandless, ^1^Stephen S. Stray, ^1^John T. Bates, ^2^Gailen D. Marshall

^1^Department of Microbiology and Immunology, and ^2^Department of Medicine, University of Mississippi Medical Center, Jackson, MS 39216

*Ritesh Tandon

**Email:**  [rtandon@umc.edu](mailto:rtandon@umc.edu)


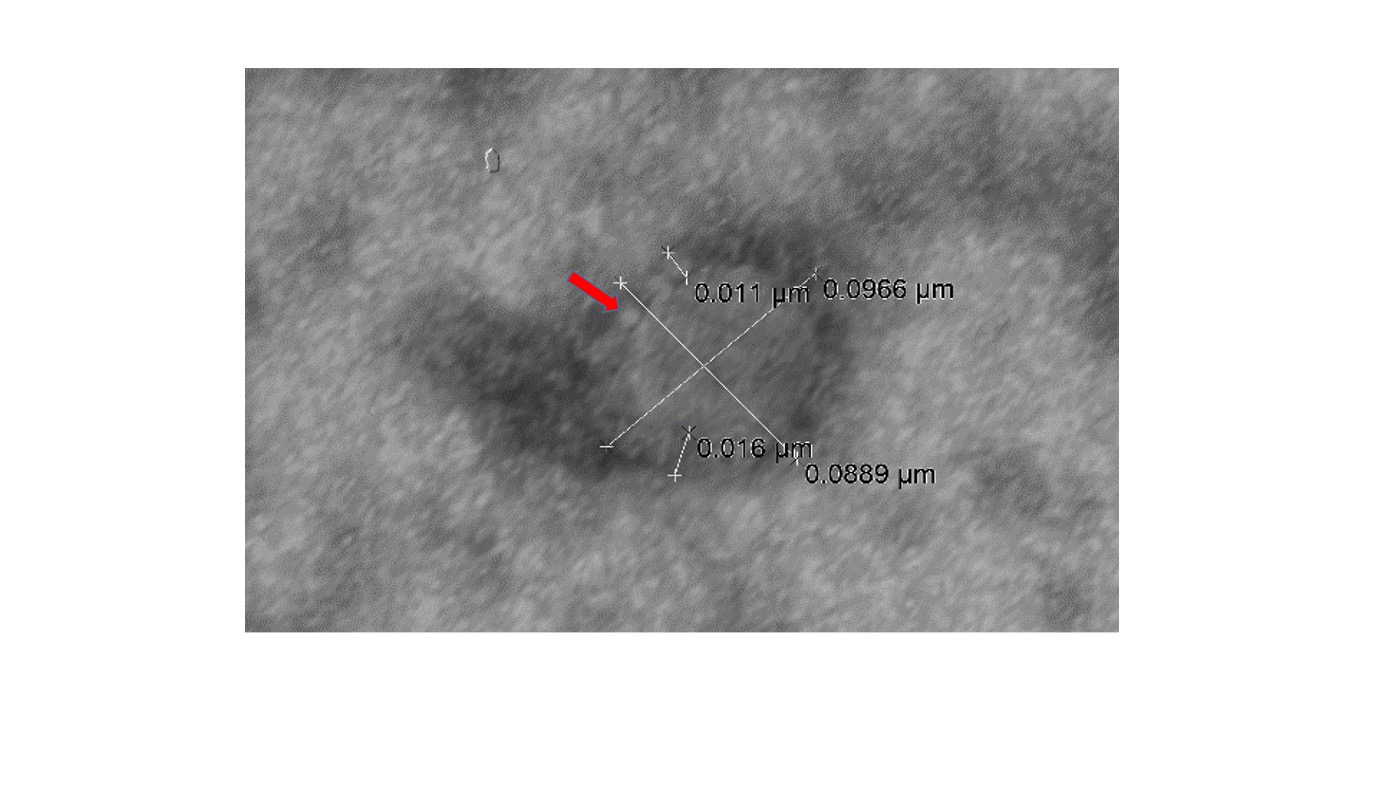


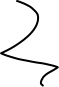

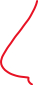


**Figure S1. Transmission electron micrograph of a negatively stained pLV-S pseudoparticle.** The spike trimer on the surface can be visualized with a length ranging from 0.011µM to 0.016 µM. The virus particle has an average diameter of 0.09275 µM.


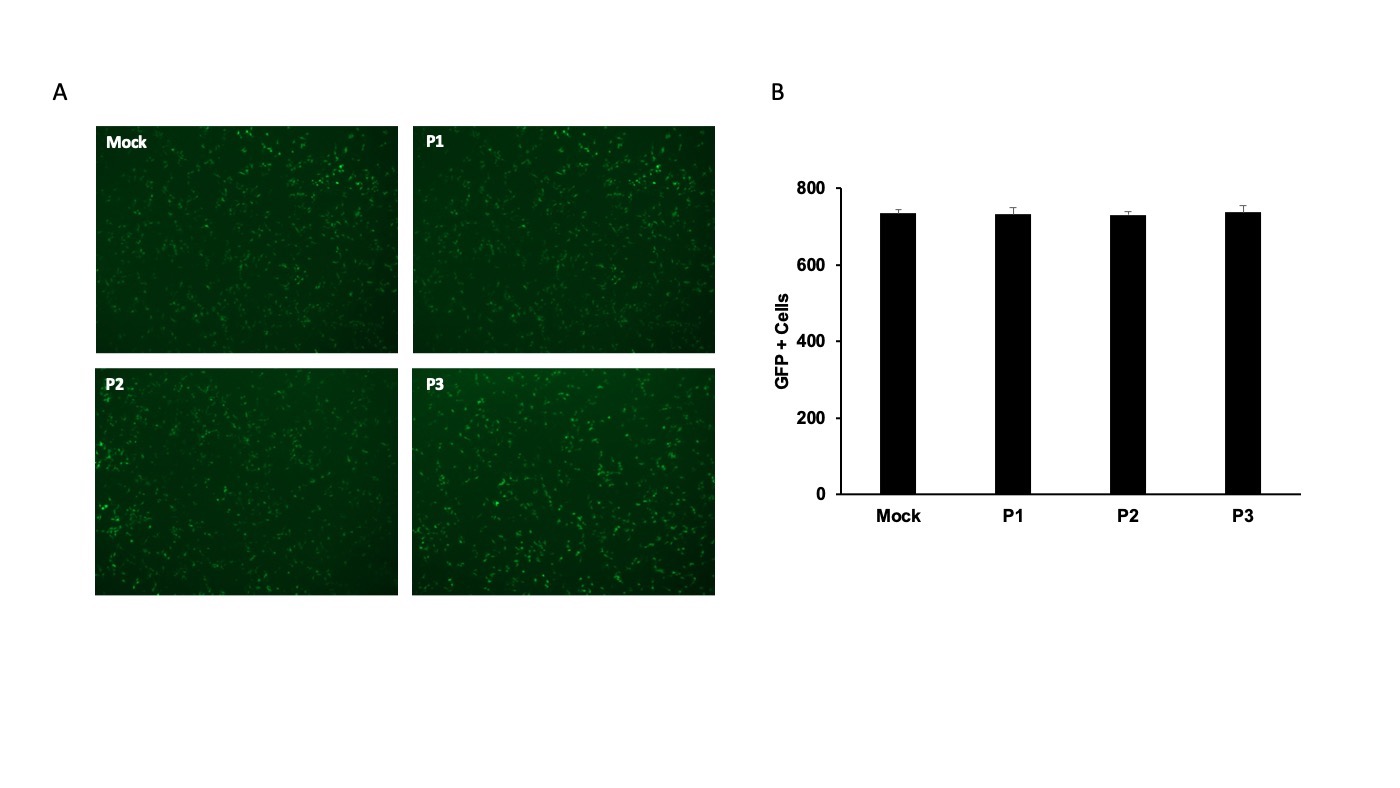


**Figure S2. VSV-G pseudotyped virus is not neutralized with SARS-CoV-2 convalescent patient serum.** 293T cells were infected with pLV-G virus incubated with serum (1:40) from three different convalescent patients. Fluorescence was recorded at 48 hours post infection. Transductions were done in triplicates and standard error of mean was plotted as error bars. A two-tailed student t-test confirmed that the differences were not significant (p >0.05).

**Figure S3. Inhibition of pLV-S using convalescent patient serum on hACE2-293T and ATCC-293T cells.** Both cell types show similar inhibition patterns.


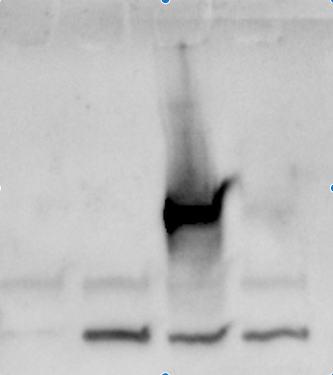


**Figure S4. Extended immunoblot from fig. 3F showing the ACE2 (dark band in the middle of the blot) and β-actin (bottom) bands.**
